# Objective Evaluation of Nasal Obstruction in Cleft Lip and Palate Patients: A Preliminary Study

**DOI:** 10.3390/jpm15090403

**Published:** 2025-09-01

**Authors:** Nicolas Pachebat, Jiad N. Mcheik, Maxime Fieux, Valentin Favier, Aurélien Binet, Xavier Dufour, Florent Carsuzaa

**Affiliations:** 1 Service d’ORL et Chirurgie Cervico-Faciale, Centre Hospitalier Universitaire de Poitiers, 2 Rue de la Milétrie, F-86000 Poitiers, France; nicolas.pachebat@chu-poitiers.fr (N.P.); xavier.dufour@chu-poitiers.fr (X.D.); 2 Faculté de Médecine, Université de Poitiers, F-86000 Poitiers, France; jiad.mcheik@chu-poitiers.fr (J.N.M.); aurelien.binet@chu-poitiers.fr (A.B.); 3 Service de Chirurgie Pédiatrique, Centre Hospitalier Universitaire de Poitiers, F-86000 Poitiers, France; 4 Laboratoire Inflammation, Tissus Epithéliaux et Cytokines, LITEC UR 155-60, Université de Poitiers, F-86000 Poitiers, France; 5 Service d’ORL et Chirurgie Cervico-Faciale, Centre Hospitalier Universitaire de Lyon Sud, F-69003 Lyon, France; maxime.fieux@chu-lyon.fr; 6 Service d’ORL et Chirurgie Cervico-Faciale, Centre Hospitalier Universitaire de Montpellier, F-34295 Montpellier, France; 7 CNRS UMR7267, Écologie et Biologie des Interactions, Université de Poitiers, F-86000 Poitiers, France

**Keywords:** cleft lip, cleft palate, nasal obstruction, rhinomanometry, personalized medicine

## Abstract

**Introduction**: Cleft lip and/or palate (CLP) is frequently associated with persistent nasal obstruction, often due to structural deformities unaddressed by primary surgical repair. While subjective assessment tools are commonly used to evaluate nasal patency, they underestimate functional impairment, particularly nasal valve collapse. This study aims to objectively evaluate nasal obstruction and identify its anatomical causes in CLP patients after primary rhinoplasty. **Methods**: We conducted an observational study involving 21 children aged 8–16 with CLP who had undergone primary cheilorhinoplasty but not secondary nasal surgery. Each participant underwent clinical evaluation, nasal endoscopy, acoustic rhinometry, and active anterior rhinomanometry (AAR), both before and after nasal decongestion. The Nasal Obstruction Symptom Evaluation (NOSE) scale was used to assess subjective symptoms. Obstructive sites were diagnosed based on established criteria combining endoscopic and functional findings. **Results**: Objective nasal obstruction was identified in 80.9% of patients, with nasal valve collapse observed in 66.7%, most commonly among unilateral and bilateral CLP subtypes. External nasal valve collapse was the predominant form (57.1%), followed by internal valve involvement (38.1%). Notably, the NOSE score did not reliably correlate with the AAR results, underlining the limitations of subjective assessment tools. Structural anomalies such as septal deviation (52.5%) and turbinate hypertrophy (23.8%) were also prevalent. **Conclusions**: This study highlights nasal valve collapse as a major, underrecognized contributor to persistent nasal obstruction in CLP patients after primary repair. Objective assessment methods like AAR and targeted endoscopy should be routinely integrated into secondary rhinoplasty planning. These findings advocate for a personalized approach to secondary nasal reconstruction in CLP patients, integrating objective functional data into surgical planning. Such strategies align with personalized medicine principles by tailoring interventions to individual anatomical and physiological characteristics.

## 1. Introduction

Cleft lip with or without cleft palate (CLP) is the most common orofacial developmental anomaly. The global prevalence of CLP approximates 7.49 per 10,000 live births [1]. Nasal deformities associated with cleft lip are known to cause nasal obstruction [2,3,4].

Surgical options regarding secondary rhinoplasty for these patients are significantly variable, with numerous techniques described in the literature [5,6]. Surgical approaches aim to restore anatomy as physiologic as possible in patients with CLP. However, the outcomes of secondary rhinoplasty remain variable and challenging to predict [5,6]. This variability can be attributed to multiple factors, including the heterogeneity of cleft severity, differences in surgical timing and techniques, and the lack of standardized preoperative functional assessment. Additionally, secondary nasal surgeries are often guided more by aesthetic considerations than by quantifiable functional indicators. As such, persistent nasal dysfunction is common even in patients with morphologically satisfactory outcomes. Recent advances in objective functional diagnostics have allowed for a more granular analysis of airflow dynamics and nasal architecture, offering a pathway toward evidence-based, individualized surgical planning. Their variability may explain why, despite multiple surgeries, adults with CLP continue to report nasal obstruction as a major complaint. A recent systematic review observes that 20% to 60% of patients experience nasal obstruction after secondary rhinoplasty [7]. The functional implications of cleft-related nasal deformities extend beyond what is visible during routine clinical evaluation. Despite advances in surgical repair, long-term follow-up data indicate that many patients continue to experience substantial airflow limitation, particularly under physical exertion or during sleep. These persistent symptoms suggest that current assessment protocols and surgical criteria may not adequately capture the dynamic components of nasal obstruction. The lack of consensus on standardized functional assessment tools has hindered the development of robust comparative studies. Moreover, the dynamic nature of nasal airflow—affected by subtle movements of the external and internal nasal valves—poses additional diagnostic challenges. Consequently, there is a growing need to reevaluate diagnostic paradigms and surgical planning through a more functional lens. This shift toward objective, individualized assessment is central to refining the long-term care of CLP patients within a personalized medicine framework.

Current methods for assessing nasal obstruction are mainly subjective and may fail to adequately address underlying structural deformities. These include deviated nasal septum, internal or external nasal valve collapse—common sequelae of both the congenital cleft and prior surgical interventions. Moreover, the altered nasal anatomy in CLP patients often results in complex three-dimensional distortions that cannot be fully captured by patient-reported outcomes or simple endoscopic evaluation. Objective assessment tools such as rhinomanometry, acoustic rhinometry, or imaging-based airway analysis may therefore offer more accurate insights into the degree and cause of nasal obstruction.

A deeper understanding of these structural causes is essential to enhance surgical techniques and improve patient outcomes. Incorporating objective functional assessments may help standardize outcome measures and refine surgical strategies for this unique patient population. This growing need for measurable, reproducible evaluation criteria has driven recent interest in combining functional and anatomical data to optimize care. Beyond its anatomical and functional implications, CLP presents a significant global health challenge with marked geographical and socioeconomic disparities. In low-resource settings, limited access to timely surgical intervention often exacerbates both functional deficits and psychosocial burdens. The variability in healthcare infrastructure means that many patients in underserved regions experience delayed or incomplete repairs, further compounding nasal airway dysfunction. Consequently, the pursuit of objective functional evaluation is not only a matter of refining surgical outcomes but also of addressing healthcare inequities by enabling standardized, reproducible assessments that transcend cultural and regional differences. In many low-resource settings, access to diagnostic tools such as rhinomanometry or high-resolution imaging remains limited, forcing clinicians to rely almost exclusively on subjective evaluations. This reliance increases the risk of underdiagnosing dynamic obstructions and delays corrective interventions. By contrast, in high-income countries, the wider availability of such technologies enables not only more accurate surgical planning but also standardized longitudinal follow-up. Addressing these disparities is essential to developing simplified, reproducible diagnostic protocols that can be adapted and implemented across diverse healthcare environments, thereby promoting equitable access to functional assessment and care for CLP patients.

The aim of our study was to objectively assess the prevalence of nasal obstruction in patients with CLP after primary rhinoplasty and to investigate its underlying structural causes. Personalized medicine emphasizes tailoring treatments to individual characteristics, and in the context of CLP, this involves adapting surgical strategies to the specific anatomical deformities and functional impairments of each patient. By integrating objective diagnostic tools, our approach aligns with this paradigm, aiming to deliver more effective and patient-specific surgical care.

## 2. Methods

We conducted an observational study at a university hospital center on patients aged 8 to 16 years with CLP who had undergone primary surgery (cheilorhinoplasty according to Millard) but not secondary rhinoplasty. This age range was selected because nasal and facial structures have reached a degree of postoperative stability, reducing the influence of early growth changes, while still allowing for the identification of functional impairments that may guide intermediate surgical interventions before final skeletal maturity. The study was approved by an institutional ethics committee, and all patients (or their legal representatives) provided their consent.

To ensure that nasal function was assessed at a time sufficiently distant from the initial surgery, nasal patency assessment was performed at least four years after the last facial surgery. This interval allowed for stabilization of the surgical results and reduced the influence of short-term healing processes. Patients with an additional nasal or pharyngeal disease were excluded in order to isolate the effects of the cleft-related anatomy from other confounding respiratory conditions. Patients with genetic syndromes or multiple malformations were excluded to ensure a homogeneous study population, minimizing variability related to syndromic features or complex craniofacial anomalies.

CLP were classified in three groups: isolated cleft palate (ICP), including any type of cleft of the palate without a lip cleft; unilateral cleft lip and palate (UCLP), including any type of unilateral cleft affecting at least the lip and alveolar ridge; and bilateral clef lip and palate (BCLP), including any type of bilateral cleft affecting at least the lip and alveolar ridge.

The presence of nasal symptoms was assessed with the visual analogic scale, and quality of life was measured using the Nasal Obstruction Symptom Evaluation (NOSE) questionnaire, a validated instrument for evaluating the functional impact of nasal obstruction on daily living [8]. Each patient underwent a standardized assessment with 1/ oro-facial examination; 2/ fiberoptic nasal endoscopy; and 3/ objective nasal obstruction measurements applying active anterior rhinomanometry (AAR) and acoustic rhinometry (AR).

The AAR and AR measurements were performed using the Rhino-sys instrument (Otopront, Germany) before and after applying a nasal vasoconstrictor with 5% xylocaine with naphazoline, thereby ensuring no contraindications. Endoscopic examinations were carried out with a 2.7 mm 30° rigid endoscope. All measurements were conducted by the same practitioner to ensure inter-operator consistency. The NOSE questionnaire was administered under the supervision of a research assistant to ensure standardized interpretation by pediatric patients. An increase in unilateral resistance > 0.6 Pa·mL^−1^·s in AAR was considered significant for nasal obstruction.

Table 1 summarizes the diagnostic criteria used to identify specific anatomical sites of nasal obstruction in patients with cleft lip and/or palate, combining clinical examination findings with active anterior rhinomanometry (AAR) and acoustic rhinometry (AR) results. For septal deviation, diagnosis required observation of a deviated septum during fiberoptic nasal endoscopy, an ipsilateral nasal airway resistance > 0.6 Pa·mL^−1^·s on AAR, and no significant improvement after topical vasoconstrictor administration. For inferior turbinate hypertrophy, diagnosis required swollen turbinates visualized on endoscopy, a nasal airway resistance > 0.6 Pa·mL^−1^·s on AAR, and a decrease in resistance ≥ 25% after vasoconstrictor application. Alternatively, a ≥25% increase in nasal volume at 2–5 cm from the nostril (MCA2) on AR after vasoconstrictor was also accepted. For nasal valve collapse, diagnosis required visible collapse of the nasal wall during moderate inspiration, subjective improvement with the modified Cottle maneuver, and a positive Fried’s test on AAR [9]. Internal nasal valve collapse was diagnosed when nasal valve collapse was associated with a positive Hysteresis test on AAR, whereas external nasal valve collapse required valve collapse with a negative Hysteresis test [10]. This structured approach ensured standardized, reproducible identification of obstruction sites, combining both anatomical and functional criteria to improve diagnostic accuracy [11]. The static or dynamic nature of the collapse was evaluated during the clinical examination. To further enhance consistency and data comparability, all evaluations were performed under standardized environmental conditions and within a single clinical setting. Rhinomanometry and acoustic rhinometry protocols strictly followed established procedural guidelines, ensuring high reproducibility. Particular attention was given to patient cooperation and positioning, both of which are known to significantly influence functional measurements in pediatric populations. Moreover, the prospective nature of the data collection minimized recall bias and allowed for a more reliable correlation between clinical findings and physiological measurements. These methodological considerations support the robustness of our findings and their relevance to similar clinical settings.

Statistics: Numerical variables were expressed as median with interquartile range, and categorical variables as absolute and relative frequencies (%). Groups were defined according to the type of cleft. As this was an exploratory preliminary study, the sample size was determined by the number of eligible patients meeting the strict inclusion criteria within the study period. Given the exploratory design and limited eligible population, no a priori sample size calculation was performed; analyses were considered hypothesis-generating. Normality was assessed using the Shapiro–Wilk test (NOSE: *p* = 0.001; AAR: *p* = 0.386). Spearman’s rho correlation for nonparametric data to determine the degree of association between two numerical variables, the rhinomanometric data and the subjective sensation of nasal patency (NOSE scores), was calculated. Patients with missing data were not excluded from the analysis. Statistical analyses were performed using R software (v. 4.2.2, R Foundation for Statistical Computing, Vienna, Austria, www.r-project.org).

## 3. Results

Twenty-three consecutive patients with a CLP were assessed. Two patients were excluded due to differential diagnosis of nasal obstruction: one presented with an obstructive nasal polyp, and another had undergone an obstructive velopharyngoplasty procedure, both of which could independently affect nasal airflow and confound the interpretation of results related to CLP-related obstruction. The population characteristics are detailed in Table 2. There was a slight female predominance with 57.1% (12/21), and the median age was 11 years (range: 8–16). The three cleft subtypes were relatively evenly distributed, with a slight predominance of UCLP in 38.1% patients (8/21), followed by BCLP in 33.3% patients (7/21) and ICP in 28.6% patients (6/21). Regarding primary surgical techniques for palatoplasty, four techniques were used, with Sommerlad and Veau–Wardill–Kilner being the most common (42.9% each, 9/21 patients). Orthodontic treatment with a quadhelix was reported in 57.1% (12/21) of patients. No patient had undergone surgical maxillary distraction. Intermediate rhinoseptoplasty was performed in 38.1% of patients (8/21), and 33.3% (7/21) had alar revision procedures. The median NOSE score was 2 [0–6], indicating mild perceived nasal obstruction. Significant unilateral or bilateral obstruction was reported by 42.9% of patients (9/21), including 33.3% (7/21) who experienced nasal breathing difficulty during physical exercises. Sleep disturbances were present in 52.4% of cases (11/21). Exclusive mouth breathing was observed in 33.3% of patients (7/21), while optimal nasal breathing was noted in 28.6% patients (6/21).

**Figure 1:**
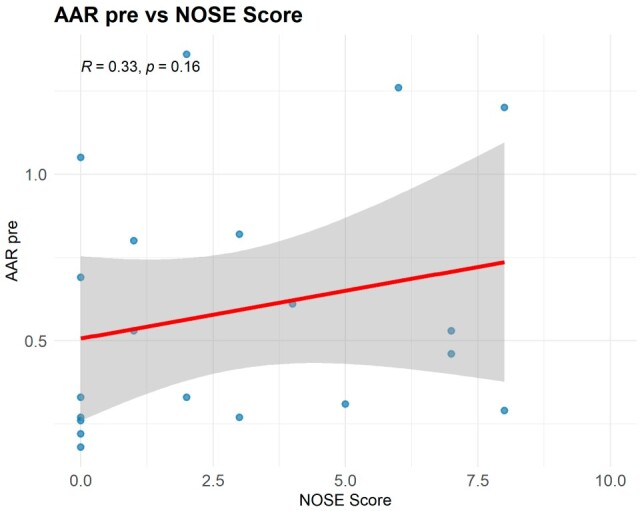
Scatter plots of NOSE vs. AAR values (pre-decongestion).

AAR revealed that 80.9% of patients (17/21) presented with significant nasal obstruction, at least unilaterally. The median NOSE score was 2 [0;6] and was poorly correlated with the severity of nasal obstruction on AAR. Among obstructive sites, nasal valve collapse was predominant, observed in 66.7% of patients (14/21). More specifically, 57.1% (12/21) of patients exhibited external nasal valve collapse, and 38.1% (8/21) showed signs of internal nasal valve involvement. In addition, a symptomatic deviated septum was identified in 52.5% of patients (11/21), while 23.8% (5/21) exhibited bilateral inferior turbinate hypertrophy. Nasal valve collapse was observed only in patients with UCLP and BCLP (Table 2). A correlation analysis between NOSE scores and AAR findings showed a weak association (Spearman correlation coefficient = 0.33; 95%CI [−0.50; 0.57]; *p* = 0.16; post hoc power for r = 0.5 = 55.6%; for observed effect ≈ 5%), indicating that subjective symptoms alone are not a reliable predictor of objective nasal obstruction in this population. Figure 1 illustrates the absence of a consistent linear trend between NOSE and AAR values.

## 4. Discussion

Our study reveals a high prevalence of nasal valve collapse among patients with cleft lip who have undergone a primary rhinoplasty procedure. This finding highlights a frequently underestimated cause of persistent nasal obstruction in this patient population. Despite initial reconstructive efforts, in these cases, the lower lateral cartilages are often asymmetrical or misplaced, contributing significantly to compromised nasal function and aesthetic disharmony [12,13]. The high prevalence of valve involvement (66.7%) we observed aligns with evidence suggesting that the valve—particularly the external valve—is a key determinant of nasal resistance in CLP patients. Few studies have explicitly reported prevalence figures for valve collapse in this population; however, a recent systematic review on functional improvement after secondary rhinoplasty underscores the importance of valve procedures in functional restoration [14]. Very recent work by Wang et al. provides a morphologic analysis of 137 patients with operated CLP nasal deformity, detailing endonasal alterations likely to increase resistance—findings consistent with the valve dominance observed in our study [15]. The low correlation between NOSE and AAR (r = 0.22) supports the notion that subjective tools alone do not fully capture the dynamic components of obstruction (internal/external valve). While the NOSE scale remains a validated and widely used instrument for subjective symptom assessment, its poor correlation with objective rhinomanometric findings in our cohort raises important concerns about its sole use in surgical planning. Patient-reported outcomes are inherently influenced by individual pain thresholds, adaptation over time, and psychosocial factors such as body image perception. This reinforces the need for a dual evaluation strategy—where subjective scales are complemented by reproducible objective measurements—to ensure that functional impairment is neither underestimated nor overlooked.

A collapse of the columella ipsilateral to the cleft is the most common residual deformity, leading to external nasal valve collapse. This deformity is frequently observed during clinical evaluation and endoscopic examination, indicating a structural weakness in the nasal framework. Due to insufficient release of the alar cartilage, which may not have been adequately addressed during the primary surgery, the dome is pushed downward by the caudal end of the triangular cartilage or the septum. This displacement compromises the integrity of the nasal valve area, which is essential for maintaining unobstructed airflow. The collapse of the dome may be associated with deformation of the nasal vestibule, which is lifted by the posterior rotation of the lower border of the lower lateral cartilage, causing a narrowing of the internal nasal valve [14]. These anatomical alterations illustrate the complexity of nasal dynamics in patients with CLP and underline the limitations of subjective evaluation tools in identifying such intricate deformities [16,17].

Persistent nasal obstruction in CLP patients may have implications that extend beyond immediate respiratory discomfort. Chronic mouth breathing, often adopted as a compensatory mechanism, can influence craniofacial growth patterns, contribute to malocclusion, and exacerbate orthodontic challenges. Furthermore, impaired nasal breathing has been associated with reduced exercise tolerance, diminished sleep quality, and increased risk of pediatric obstructive sleep apnea. The high prevalence of valve collapse identified in our cohort therefore warrants early detection and timely intervention to mitigate these downstream effects, particularly in growing children. Beyond its functional implications, persistent nasal obstruction and visible nasal asymmetry can have a profound psychosocial impact on children and adolescents with CLP. Peer perception, self-image, and social participation are often influenced by facial appearance and the ability to breathe comfortably, particularly during speech or physical activities. Functional impairments such as chronic mouth breathing can alter speech resonance, potentially drawing unwanted attention and exacerbating social anxiety. Addressing both the aesthetic and functional aspects of nasal reconstruction is therefore not only a matter of improving airflow but also of enhancing overall quality of life and psychosocial well-being. This dual focus is essential for optimizing long-term rehabilitation outcomes in this patient population.

In patients with CLP, a staged surgical strategy that balances functional improvement with preservation of nasal growth could be proposed. When significant obstruction persists despite primary rhinoplasty and orthodontic treatment, and preferably objectively confirmed by active anterior rhinomanometry or acoustic rhinometry, intermediate functional procedures—such as external valve stabilization with alar or batten grafts, or internal spreader graft placement—are considered between 8 and 12 years of age. Definitive rhinoplasty is deferred until completion of nasal growth, typically around 15–16 years in females and 16–17 years in males, to allow for final correction of symmetry and optimization of valve dynamics. This approach minimizes the risk of growth disturbance while ensuring that surgical indications are based on robust objective criteria, including rhinometric data and dynamic endoscopic assessment [12,13,14].

Our study suggests that an objective assessment of nasal obstruction in patients operated on for CLP must include a precise nasal valve collapse assessment, with emphasis on both external and internal components. This comprehensive evaluation enables the personalization of secondary rhinoplasty procedures, guiding surgeons toward targeted structural corrections rather than relying solely on aesthetic refinement or symptomatic reports.

Surgical treatment of nasal valve collapse remains the gold standard [18]. Effective management often requires technically nuanced interventions tailored to the specific type and location of collapse. For external valve collapse, alar batten grafts or lateral crural flip flap are commonly used [7,19], both techniques aiming to reinforce or reposition weakened cartilage structures. Internal valve collapses are treated by expanding the cross-sectional area with spreader grafts or flaps as well as butterfly grafts, which serve to widen the narrowest part of the nasal airway and improve stability during inspiration [20]. Alternatively, a resorbable implant such as LATERA^®^ (Stryker Corp., Kalamazoo, MI, USA) may strengthen the cartilage, offering a minimally invasive solution; however, its use in cleft patients is unstudied, and its efficacy in anatomically complex cases remains to be determined through focused clinical trials [21].

The main limitation of this study is inherent to its single-center design, with all patients treated by a single surgeon. While this uniformity ensures consistency in surgical technique and postoperative assessment, it also limits the generalizability of the findings. Multicenter studies are required to corroborate these findings, ideally encompassing diverse surgical teams and patient populations, to validate the observed associations and support broader clinical recommendations. The relatively small sample size limits statistical power and generalizability. Nevertheless, the uniform surgical history and standardized evaluation strengthen the internal validity of the findings. The study is underpowered to detect small-to-moderate correlations: post hoc power was ~55–59% for a moderate effect (r = 0.5) but only ~5% for the observed weak correlations. Therefore, our findings must be interpreted with caution. Furthermore, subgroup analyses (BCLP, UCLP, ICP) are descriptive only and underpowered; they should be regarded as exploratory rather than confirmatory. Moreover, although 52.4% of patients reported sleep disturbances, no polysomnography or standardized nocturnal oximetry was performed. The clinical impact of nasal obstruction on sleep in this cohort therefore remains inferential. Future studies correlating AAR/AR with polysomnographic indices (AHI, oxygen desaturation) are warranted.

Optimal management of CLP patients also requires a coordinated multidisciplinary approach. Functional outcomes are influenced not only by surgical technique but also by factors involving orthodontics, speech therapy, and otolaryngology, highlighting the need for ongoing collaboration across specialties. For instance, orthodontic devices such as the quadhelix may alter nasal architecture and affect resistance measurements obtained through rhinomanometry. Similarly, close coordination with speech therapists enables clinicians to better assess the impact of nasal obstruction on velopharyngeal function and respiratory patterns, especially during sleep or physical activity. A multidisciplinary team can thus offer a more comprehensive diagnostic perspective and anticipate the need for therapeutic adjustments, including postoperative modifications. By enhancing these collaborative efforts, cleft care centers can improve diagnostic accuracy, guide individualized interventions, and optimize both short- and long-term patient outcomes.

Given the heterogeneity of cleft phenotypes and surgical histories, future research should prioritize multicenter prospective studies with larger sample sizes to validate the diagnostic algorithms proposed here. Collaboration between craniofacial surgeons, otolaryngologists, orthodontists, and biomedical engineers will be essential to develop integrated assessment protocols that are both clinically feasible and scientifically robust. Such collaborative networks could also serve as platforms for longitudinal studies assessing the durability of functional improvements after secondary interventions, thereby informing best practice guidelines in CLP nasal reconstruction.

This personalized evaluation model allows surgeons to design interventions based on the unique anatomical and functional profile of each patient rather than relying solely on cleft type or standard techniques. This individualized strategy could lead to more predictable surgical outcomes, reduced symptom recurrence, and better alignment with the goals of precision healthcare. Emerging technologies may further enhance this individualized approach. For instance, computational fluid dynamics (CFD) simulations based on patient-specific imaging could allow for preoperative visualization of nasal airflow patterns and potential zones of collapse [22]. These models offer a promising adjunct to clinical examination by enabling simulation of various surgical scenarios and their functional consequences. Additionally, 3D endoscopic imaging and machine-learning algorithms may support the development of predictive tools to stratify surgical candidates based on anatomical and physiological parameters [23]. Integrating these innovations into clinical practice could significantly improve decision-making and optimize outcomes in cleft-related rhinoplasty.

## 5. Conclusions

In patients with CLP, we have highlighted a high prevalence of nasal valve collapse, despite primary rhinoplasty. This finding emphasizes the need for systematic nasal valve assessment, as anatomical distortions often persist and contribute to long-term nasal obstruction.

Combined with clinical examination, objective evaluation of the nasal valve could be integrated into the planning of secondary rhinoplasty. This would support a more personalized surgical approach, targeting the specific structural causes of obstruction. By tailoring interventions to each patient’s anatomy and function, outcomes may be optimized both functionally and aesthetically.

In the future, integrating objective data from rhinomanometry with patient-specific computational modeling could enable the prediction of the functional impact of each surgical maneuver. Such predictive approaches, combining artificial intelligence with 3D imaging, may provide powerful tools to design individualized interventions and reduce the need for revision procedures. In parallel, simplified measurement protocols, applicable in routine outpatient settings or low-resource environments, should be developed to democratize access to reliable functional assessment. These strategies would contribute to improving both equity and quality of care in CLP nasal reconstruction.

## Figures and Tables

**Table 1:** Diagnosis of obstructive sites using clinical examination and active anterior rhinomanometry (AAR).

Obstructive Sites	Diagnosis Method
Septal deviation	Deviated septum in fiberoptic nasal endoscopy (clinic) AND Ipsilateral air resistance > 0.6 Pa·mL^−1^·s (AAR) AND Lack of improvement after vasoconstrictor (AAR)
Inferior turbinate hypertrophy	Swollen turbinate in fiberoptic nasal endoscopy AND Air resistance > 0.6 Pa·mL^−1^·s (AAR) AND Decrease in air resistance ≥ 25% after nasal vasoconstrictor (AAR) OR Increase in nasal volume of at least 25% between 2 and 5 cm (MCA2) from the nostril after nasal vasoconstrictor (AR)
Nasal valve collapse	Nasal wall collapse during moderate inspiration AND Subjective improvement with modified Cottle maneuver AND Positive Fried’s test (AAR)
Internal nasal valve collapse	Nasal valve collapse AND Positive Hysteresis test (AAR)
External nasal valve collapse	Nasal valve collapse AND Negative Hysteresis test (AAR)

**Table 2:** Nasal characteristics depending on cleft type. IQR: interquartile range; NOSE: Nasal Obstruction Symptom Evaluation; AAR: active anterior rhinomanometry.

	Bilateral Cleft Lip and Palate *n* = 7	Unilateral Cleft Lip and Palate *n* = 8	Isolated Cleft Palate *n* = 6
Gender (male), *n* (%)	4 (57.1)	3 (37.5)	2 (33.3)
Median age, years (IQR)	12 (11;14)	10.5 (9;14)	10.5 (9.25;11.75)
Initial reconstruction surgery type			
Primary Cheilorhinoplasty			
Millard, *n* (%)	7 (100)	8 (100)	0 (0)
Intravelar Veloplasty			
Sommerlad, *n* (%)	3 (42.9)	3 (37.5)	3 (50)
Veau–Wardill–Kilner, *n* (%)	4 (57.1)	4 (50)	1 (16.7)
Von Langenbeck, *n* (%)	0 (0)	0 (0)	1 (16.7)
Furlow, *n* (%)	0 (0)	0 (0)	1 (16.7)
Median delay since last nasal surgery, years (IQR)	9 (8;10.75)	6.4 (4.25;9.25)	NA
Median NOSE (IQR)	3 (1.5;6.5)	3.5 (0.75;5.75)	0 (0;1.5)
Symptoms			
Nasal obstruction EN ≥ 5, *n* (%)	3 (42.9)	4 (50)	2 (33.3)
Rhinorrhea EN ≥ 5, *n* (%)	0 (0)	1 (12.5)	0 (0)
Sleep disorders, *n* (%)	5 (71.4)	5 (62.5)	1 (16.7)
Nasal breathing discomfort on effort, *n* (%)	3 (42.9)	3 (37.5)	1 (16.7)
Exclusive mouth breathing, *n* (%)	4 (57.1)	3 (37.5)	0 (0)
Optimal nasal breathing, *n* (%)	1 (14.3)	0 (0)	5 (83.3)
Nasal obstruction in AAR, *n* (%)	7 (100)	8 (100)	2 (33.3)
Obstructive Sites	2 (28.6)	2 (25)	0 (0)
Nasal valve collapse, all types, *n* (%)	0 (0)	7 (87.5)	0 (0)
Internal nasal valve collapse, *n* (%)	5 (71.4)	5 (62.5)	0 (0)
Dynamic, *n* (%)	4 (57.1)	2 (25)	0 (0)
Static, *n* (%)	1 (14.3)	7 (87.5)	0 (0)
External nasal valve collapse, *n* (%)	3 (42.9)	4 (50)	1 (16.7)
Dynamic, *n* (%)	0 (0)		1 (16.7)
Static, *n* (%)		0 (0)	0 (0)
Septal deviation, *n* (%)	8 (100)	0 (0)	0 (0)
Bilateral turbinate hypertrophy, *n* (%)	6 (75)	2 (25)	0 (0)

## Data Availability

The data presented in this study are available on request from the corresponding author due to legal reasons.

## References

[1] Tanaka S.A., Mahabir R.C., Jupiter D.C., Menezes J.M. (2012). Updating the Epidemiology of Cleft Lip with or without Cleft Palate. Plast. Reconstr. Surg..

[2] Zhang R.S., Lin L.O., Hoppe I.C., Jackson O.A., Low D.W., Bartlett S.P., Swanson J.W., Taylor J.A. (2019). Nasal Obstruction in Children with Cleft Lip and Palate: Results of a Cross-Sectional Study Utilizing the NOSE Scale. Cleft Palate Craniofacial J..

[3] Sobol D.L., Allori A.C., Carlson A.R., Pien I.J., Watkins S.E., Aylsworth A.S., Meyer R.E., Pimenta L.A., Strauss R.P., Ramsey B.L. (2016). Nasal Airway Dysfunction in Children with Cleft Lip and Cleft Palate: Results of a Cross-Sectional Population-Based Study, with Anatomical and Surgical Considerations. Plast. Reconstr. Surg..

[4] Warren D.W., Hairfield W.M., Dalston E.T., Sidman J.D., Pillsbury H.C. (1988). Effects of Cleft Lip and Palate on the Nasal Airway in Children. Arch. Otolaryngol. Head Neck Surg..

[5] Nicol M., De Boutray M., Captier G., Bigorre M. (2022). Primary cheilorhinoseptoplasty using the Talmant protocol in unilateral complete cleft lip: Functional and aesthetic results on nasal correction and comparison with the Tennison–Malek protocol. Int. J. Oral Maxillofac. Surg..

[6] Pinto V., Piccin O., Burgio L., Summo V., Antoniazzi E., Morselli P.G. (2018). Effect of early correction of nasal septal deformity in unilateral cleft lip and palate on inferior turbinate hypertrophy and nasal patency. Int. J. Pediatr. Otorhinolaryngol..

[7] Yuan J., An Y. (2024). Improvement in nasal airway obstruction after secondary rhinoplasty for cleft lip: A systematic review. J. Plast. Reconstr. Aesthetic Surg..

[8] Stewart M.G., Witsell D.L., Smith T.L., Weaver E.M., Yueh B., Hannley M.T. (2004). Development and Validation of the Nasal Obstruction Symptom Evaluation (NOSE) Scale1. Otolaryngol. Head Neck Surg..

[9] Maalouf R., Bequignon E., Devars Du Mayne M., Zerah-Lancner F., Isabey D., Coste A., Louis B., Papon J.-F. (2016). A functional tool to differentiate nasal valve collapse from other causes of nasal obstruction: The FRIED test. J. Appl. Physiol..

[10] Gagnieur P., Fieux M., Louis B., Béquignon E., Bartier S., Vertu-Ciolino D. (2022). Objective diagnosis of internal nasal valve collapse by four-phase rhinomanometry. Laryngoscope Investig. Otolaryngol..

[11] Petitjean M., Béquignon É., Fieux M., Louis B., Zerah F., Coste A., Bartier S. (2022). COVID-19 pandemic: Do surgical masks impact respiratory nasal functions?. Int. Forum Allergy Rhinol..

[12] Dissaux C., Grollemund B., Bodin F., Picard A., Vazquez M.-P., Morand B., James I., Kauffmann I., Bruant-Rodier C. (2016). Evaluation of 5-year-old children with complete cleft lip and palate: Multicenter study. Part 2: Functional results. J. Cranio-Maxillofac. Surg..

[13] Talmant J.C. (2000). Current Trends in the Treatment of Bilateral Cleft Lip and Palate. Oral Maxillofac. Surg. Clin. N. Am..

[14] Hsieh T., Gengler I., Tollefson T.T. (2024). Rhinoplasty for Patients with Cleft Lip-Palate. Otolaryngol. Clin. N. Am..

[15] Wang Y., Zhang Z., Sun W., Song T., Yin N., Wang Y. (2025). Morphologic Analysis of Nasal Airway in 137 Patients with Operated Cleft Lip Nasal Deformity. J. Craniofacial Surg..

[16] Gelardi M., Intiglietta P., Porro G., Quaranta V.N., Resta O., Quaranta N., Ciprandi G. (2019). The role of the nasal valve in patients with obstructive sleep apnea syndrome. Acta Biomed..

[17] Chung V., Lee A.S., Scott A.R. (2014). Pediatric nasal valve surgery: Short-term outcomes and complications. Int. J. Pediatr. Otorhinolaryngol..

[18] Yarlagadda B.B., Dolan R.W. (2011). Nasal valve dysfunction: Diagnosis and treatment. Curr. Opin. Otolaryngol. Head Neck Surg..

[19] Totonchi A., Guyuron B. (2016). Alar Rim Deformities. Clin. Plast. Surg..

[20] Clark J.M., Cook T.A. (2002). The ‘Butterfly’ Graft in Functional Secondary Rhinoplasty. Laryngoscope.

[21] Kim D.H., Lee H.H., Kim S.H., Hwang S.H. (2020). Effectiveness of using a bioabsorbable implant (Latera) to treat nasal valve collapse in patients with nasal obstruction: Systemic review and meta-analysis. Int. Forum Allergy Rhinol..

[22] Yang Z., Mao M., Li Q., Ni X. (2025). The changes of upper airway flow in adolescents with maxillary cleft before and after bone grafting and orthodontic treatment: A CFD simulation study. Comput. Methods Biomech. Biomed. Eng..

[23] Yodrabum N., Chaisrisawadisuk S., Apichonbancha S., Khaogate K., Noraset T., Sakkitjarung C., Moore M.H. (2025). Artificial Intelligence and Human Expertise in Cleft Lip and Palate Care: A Comparative Study of Accuracy, Readability, and Treatment Quality. J. Craniofacial Surg..

